# Combustion Calorimetry with Fluorine: Constant Pressure Flame Calorimetry^1^

**DOI:** 10.6028/jres.064A.005

**Published:** 1960-02-01

**Authors:** G. T. Armstrong, R. S. Jessup

## Abstract

Instruments and methods have been developed and are described for the measurement of heats of reaction between fluorine and other gaseous materials. Verification of the amount of reaction of hydrogenous materials is possible. The estimated accuracy of measurements is about 0.3 percent. Lack of certainty of the magnitude of corrections to be applied for hydrogen fluoride nonideality is an important factor. The heat of formation of hydrogen fluoride is found to be −64.4 ± 0.25 kcal/mole on the basis of the reaction of fluorine with ammonia.

## 1. Introduction

For the determination of the heat of formation of a compound from its elements, the study of suitable reactions involving directly each of the individual elements is essential. In some cases the heat of formation of a given substance may be obtained by measuring the heat of a single reaction, for example,

$$C(c,\text{graphite})+O_{2}(g)\to {\text{CO}}_{2}(g).$$

In general, however, it is necessary to combine the heats of several reactions, as for example

$$\text{CO}(g)+\frac{1}{2}O_{2}(g)\to {\text{CO}}_{2}(g)$$

$$\frac{C(c,\text{graphite})+O_{2}(g)\to {\text{CO}}_{2}(g)}{C(c,\text{graphite})+\frac{1}{2}O_{2}(g)\to {\text{CO}}_{2}(g)}.$$

Since the measured heat of each reaction used in calculating the heat of formation of a given compound may be subject to unknown experimental error, confirmation of the result by essentially different procedures; i.e., by combination of the heats of different sets of reactions permits greater confidence to be placed in the value obtained. Such confirmation is especially valuable in dealing with a group of compounds for which satisfactory procedures for measurements of heats of reaction have not been established, as they have been, for example, for combustion in oxygen of carbon-hydrogen-oxygen compounds.

Fluorine compounds furnish an example of such a group. In this group many of the methods of measurement of heats of reaction are subject to great experimental difficulties, and consequently, the values obtained for the heats of such reactions are subject to relatively large uncertainties. This is particularly true for reactions involving elemental fluorine. The development of valid procedures for accurate determination of heats of reaction involvings elemental fluorine, therefore, promises valuable improvements in the reliability of heats of formation of fluorine-containing compounds. The potential usefulness of such procedures is augmented by the fact that fluorine, the most active nonmetal, reacts vigorously with most elements and with very many compounds, and thus the development of such procedures would open a wide area of possible reaction study. Finally, fluorine, as a monovalent element forms some compounds differing considerably in physical characteristics from the corresponding oxygen compounds, and thus opens up avenues of approach to compounds accessible only with difficulty by the use of oxygen.

The development of procedures for the direct utilization of fluorine as an oxidizer in combustion calorimetry has been retarded by certain obvious difficulties: the difficulty of finding suitable containers for the reaction processes, so as to avoid side reactions with the apparatus itself; the difficulty of obtaining pure fluorine; the difficulty of handling fluorine safely because of its toxicity and its corrosive effects on equipment. Despite these difficulties a limited number of thermal studies of reactions have been carried out with elemental fluorine, which have provided the present basis of the thermochemistry of fluorine compounds. See for example, studies of the reaction of fluorine with hydrogen by von Wartenberg and Fitzner [1],^2^ von Wartenberg and Schütza [2], Ruff and Menzel [3]; and the reaction of fluorine with carbon by von Wartenberg and Schütte [4]. With the exception of the latter difficult and, as was shown later, not very accurate experiment on the combustion of charcoal, all of the experiments mentioned involved fluorine as the minor constituent, a procedure which avoids part of the difficulties mentioned above but severely limits the general applicability of the reactions. Thus for example, the study of the combustion of hydrocarbons by this process would be very difficult because of the multiplicity of products. At the same time, impurities in the fluorine would react where possible, whereas they would be excluded by their lesser reactivity from reaction in an excess of fluorine.

A considerable improvement in technique and materials for handling fluorine has occurred since those pioneering investigations, made under great difficulty, so that the development of accurate methods for reaction calorimetry of fluorine is now possible and practical, either by constant-volume bomb or constant-pressure flame processes. A series of research tasks carried out at NBS during the course of several years demonstrates the feasibility, in particular, of the constant-pressure flame calorimeter employing fluorine as the oxidizing atmosphere, indicates specific aspects of some problems encountered, and delineates procedures which appear to have general applicability in the solution of these problems. The procedures are discussed in detail in later sections of this paper. It is not claimed by any means that flame combustion calorimetry with fluorine can at present give results of accuracy comparable with the oxygen flame calorimeter in systems which have been most carefully studied. The accuracy of the fluorine flame calorimeter is of the order of 0.3 percent, compared to 0.01 percent under the best circumstances for the oxygen flame calorimeter [5]. The difference should not be taken as an indication of the relative ultimate accuracy obtainable, but rather of the present relative states of development of experimental techniques for handling the reactions involved.

Some general features of fluorine combustion systems may be pointed out, providing a key to the methods which have been adopted. Hydrogenous materials and some other substances generally ignite spontaneously at room temperature in an atmosphere of fluorine. This hypergolic behavior renders initiation of reaction easy except for those substances having a long induction period (notably hydrogen itself) which may detonate because of appreciable premixing before ignition unless an ignition device is provided. On the other hand, the premixing of fuel and oxidizer is not feasible, and so a diffusion flame must be used, with some resulting problems in burner design to achieve complete combustion. The flame once initiated may be expected to continue to burn in excess fluorine even when the concentration of fuel in the entering gases is extremely small, allowing a thorough flushing of the fuel flow lines, and also permitting extension of the method to the combustion of volatile liquids which may be vaporized into a gas stream for admittance to the burner. Hydrogen, as a constituent of a fuel, rapidly and completely combines with fluorine to form hydrogen fluoride, which can readily be quantitatively absorbed and weighed to obtain a confirmation of the amount of reaction. Some specific problems also arise in the interpretation of heat measurements because of the extreme nonideality of hydrogen fluoride gas.

In the discussion which follows are given details of instruments and methods which have been used in studies of fluorine flame calorimetry at the National Bureau of Standards, a discussion of the problem of the nonideality of hydrogen fluoride, and comments on the findings of studies of the combustion in several particular systems.

## 2. Instruments and Methods

The calorimetric method used resembles in many respects the procedure described by Rossini [5] for the combustion of hydrogen and other gases in oxygen. In brief outline the process is as follows: a weighed sample of fuel is introduced at a uniform rate into a burner in which is flowing a continuous stream of fluorine in excess. The burner and the combustion chamber of which it forms a part, are submerged in a water-filled, weighed, well-stirred calorimeter inside a constant temperature enclosure (“jacket”), from which it is insulated by an airspace. Hydrogen fluoride (if formed) and perhaps other products are collected and their amounts determined. The temperature rise of the calorimeter, the energy-equivalent of the calorimeter, and the quantity of reaction determined from the measured quantities of reactants and products, together with certain corrections, are used for the calculation of the heat of reaction for unit quantity of reactant.

### 2.1. Calorimeter and Burner

**Figure 1:**
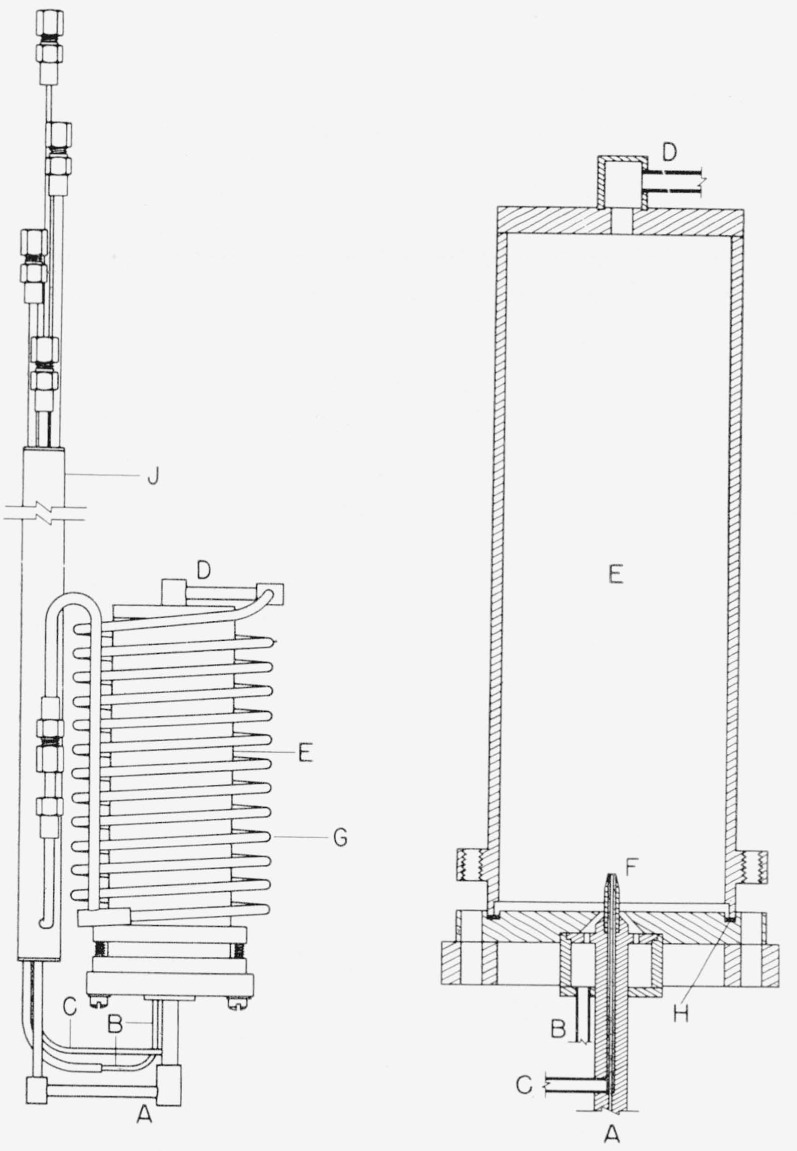
Burner for fluorine flame calorimetry *A, B*, *C*, fuel, fluorine, and helium flow lines; *D*, combustion chamber exit; *E*, burner chamber; *F*, burner tip; *G*, monel coil; *H*, Teflon gasket; *J*, heat interchanger for incoming and outgoing gases.

A diagram of the burner and combustion chamber used in the calorimetric work is shown in figure 1. The gases entering the calorimeter pass through the interchanger *J* and are then brought to the burner *F* through concentric openings. Fuel enters through copper tube *A*. Fluorine in tube *B* enters the combustion chamber *E* through an orifice in the base plate. In the combustion of hydrocarbons in fluorine in a Monel or copper burner, carbon formation is very considerable if the flame is in contact with the metal [6]. An annular stream of helium is therefore introduced through tube *C* surrounding the fuel orifice in order to prevent such contact and inhibit carbon formation. The gases leave the combustion chamber at *D* through a Monel tube wound in a coil *G* from which they pass to the interchanger *J* again and are brought to the temperature of the entering gases before leaving the calorimeter. The combustion chamber and its base plate are made of copper. A Teflon gasket *H* seals the joint between the combustion chamber body and base and permits disassembly of the chamber for inspection and cleaning.

An exploratory burner similar in general outline to the above but differing in details is used for preliminary studies. The combustion chamber of the exploratory burner has a replaceable glass window through which the flame can be observed for short periods of time and has two electrodes for ignition of the flame when necessary. If ignition of the flame by spark or hot wire is necessary, it should be anticipated that any fine wires used in the igniter will burn back to their contacts with a massive metal surface.

For calorimetric work the combustion chamber and most of the interchanger are immersed in the stirred water of a calorimeter similar to that described by Dickinson [7]. The calorimeter is in an airspace submerged in a well-stirred constant-temperature water jacket. The water jacket is kept isothermal to a few thousandths of a degree by an electronic thermo-regulator having a temperature-sensitive resistor as a sensing element. Temperatures are measured by a platinum resistance thermometer, immersed in the water surrounding the burner. Temperatures can be read reproducibly to 0.0001° C, but in these experiments the errors from other sources make temperature differences less than 0.001° C insignificant.

### 2.2. Gas Flow System

**Figure 2:**
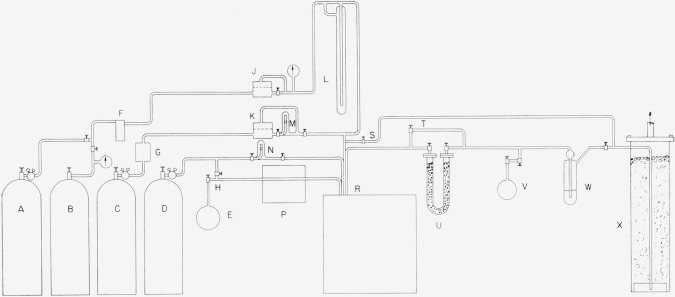
Schematic flow diagram of gases in fluorine flame calorimetry *A*, fluorine; *B, C*, helium; *D*, oxygen; *E*, fuel; *F*, hydrogen fluoride trap; *G*, drying tube; *H*, connection for purging fuel lines; *J*, *K*, mechanical flow regulators; *L*, orifice-type flowmeter for fluorine; *M, N*, float-type flowmeters; *P*, fuel flow regulator; *R*, calorimeter; *S, T*, fluorine bypass valves; *U*, hydrogen fluoride collector; *V*, gas product sample collector; *W*, bubbler; *X*, fluorine absorbing tower.

A typical diagram of gas flow lines of the combustion system is shown in figure 2, as used for a study of the combustion of methane in oxygen-fluorine mixtures [8]. Flow lines for other combustion systems may be similar but are adapted to the particular properties of the gases to be handled. Where oxygen is not desired in the combustion the specific flow lines required to introduce it are omitted. Gas supplies shown in the figure are fluorine, *A*; helium, *B* and *D*; oxygen, *C*; and fuel, *E*. Flow rates of helium, fluorine, oxygen, and other gases drawn from large tanks are regulated by small mechanical regulators as indicated at *J* and *K* which act to keep constant pressure differentials across valves immediately following the regulators. Fluorine is metered by a calibrated orifice meter *L* using Kel-F No. 1 oil as an indicating liquid. Oxygen and helium are metered by float-type flowmeters *M* and *N*. Metering and flow rate control are relatively simple for these gases because the tank pressure does not change very much during a run. The fuel, however, is drawn from a small sample bulb suitable for weighing. If it is present as a gas, a large fraction, perhaps 2/3 is drawn from the bulb in each experiment. For the most careful work it would be desirable to have the rate of temperature rise, and therefore, the rate of fuel flow constant during the combustion period. However, the large changes of pressure make the problem of obtaining constant flow rate somewhat difficult. A suitable flow rate controller had not been satisfactorily developed in time to use for the experiments thus far described, so that some sacrifice of flow rate constancy was made. In the case of a condensable gas such as ammonia, fairly good pressure and hence flow regulation is obtained by keeping the fuel as a liquid at constant temperature. Provision for a flow regulator for the fuel is shown at *P*. However, in the experiments thus far performed a needle valve is at *P*. In addition to separating the flame from the burner, helium is also used to purge the lines. Tank *B* is used to clear fluorine from the flow system as a whole in preparation for opening; tank *D* is used to carry all fuel released from bulb *E* into the burner for combustion. To this end, the fuel flow lines are made of small diameter tubing and connecting components are designed to minimize entrapment of gas. To reduce introduction of impurities into the burner to a minimum, fluorine passes through an NaF trap *F* which removes HF. In most of the experiments a similar trap (not shown) was immersed in a dry ice-alcohol bath immediately following trap *L* to remove Kel-F vapors which may be carried from the flowmeter. A drying tube G containing calcium sulfate is used to dry oxygen.

The calorimeter is shown without detail at *R*. A trap *U* in the flow line immediately following the calorimeter is packed with sodium fluoride pellets prepared in an active form for absorbing hydrogen fluoride by heating sodium bifluoride pellets to approximately 400° C in a stream of nitrogen. Trap *U* can be reactivated several times before refilling is required. This trap quantitatively absorbs hydrogen fluoride which is determined by weighing. To reduce collection of unwanted materials, gases flowing through the calorimeter bypass this trap through valve *T* except during the combustion reaction and subsequent flushing of the burner chamber.

A tee in the flow line immediately following the sodium fluoride trap *U* permits attachment of an evacuated glass bulb *V*, containing a little mercury, for collecting a sample of product gases during a run. Beyond the tee the gases pass through a bubbler *W* of glass containing Kel–F oil. The purpose of the bubbler is to prevent possible suction of outside gases into the sample bulb when drawing a sample of combustion products. The gases finally pass into a fluorine absorbing tower *X* of Monel tubing with a welded Monel bottom, packed with granular soda-lime. The gases pass upward through the packing. The soda-lime tower becomes hot to the touch during an experiment, the position of the hot spot serving to indicate the degree of exhaustion of the packing. The effluent gases are released into a laboratory hood, but no odor of fluorine is ordinarily detected from this source. A bypass is provided from directly before the calorimeter to the soda-lime absorber, valve *S*, permitting fluorine to be diverted from the calorimeter and traps. This precaution was found to be desirable to permit quick cleaning up of the system in case of line blockages, which were not infrequent.

The flow lines may be of copper but are preferably of heavy-wall Monel tubing, despite the greater difficulty of bending the latter. Connections are silver soldered where solder is necessary. A commercial Monel compression-type fitting and commercial Monel valves with Teflon packing have been found satisfactory in some flow lines involving fluorine. Where small valve volume is desirable as in the lines through which fuel and products flow, Monel or stainless steel valves [9] of a design due to H. F. Stimson of NBS have been found satisfactory. Connections to these valves and in other places where easy disassembly is desirable are made with small cone fittings.

### 2.3. Analysis of Fluorine

**Figure 3:**
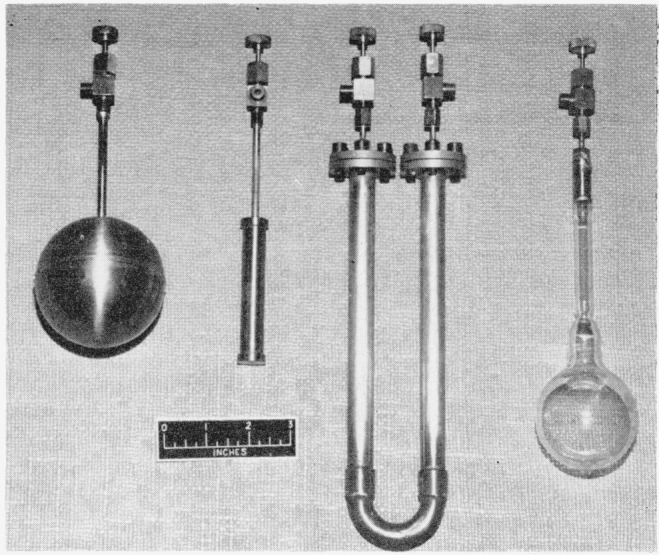
Vessels used in fluorine flame calorimetry Left to right: Monel fuel container for noncondensable gases, stainless steel fuel container for condensable gases, hydrogen fluoride collector, bulb for fluorine analysis.

**Figure 4:**
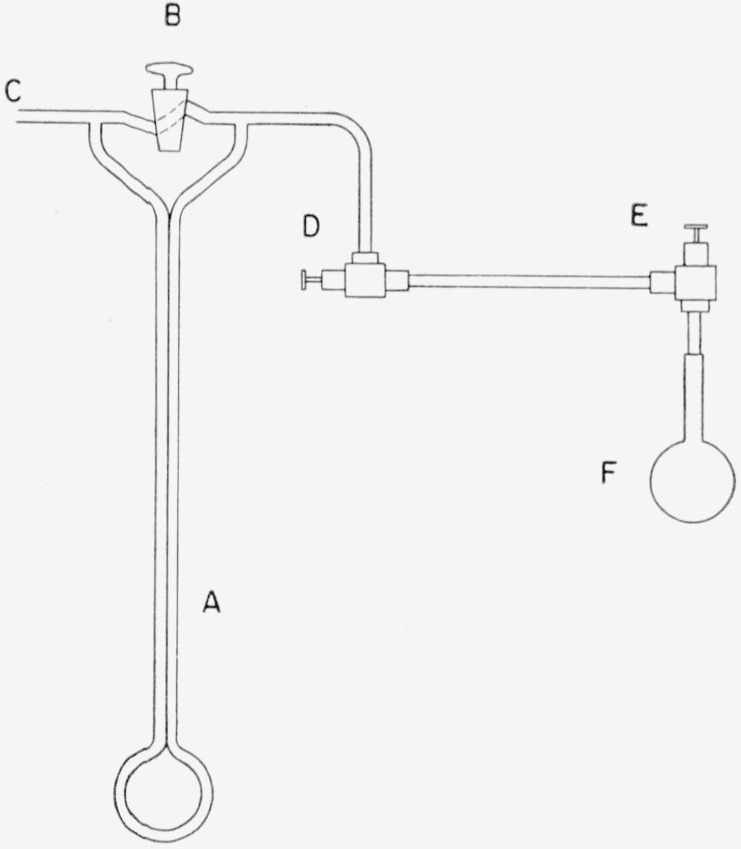
Fluorine impurity tester *A*, oil filled manometer; *R*, valve; *C*, vacuum source; *D, E*, metal valves; *F*, fluorine analysis bulb.

Because of the difficulty of obtaining very pure fluorine, analysis is desirable in order to be sure whether any factor modifying the experiment is present. For the accuracy required here fluorine of reasonably good (97 to 99 percent) purity can be quickly analyzed by absorption in mercury and measurement of the residual pressure of nonreactive gases [10, 11]. For this purpose a 50-ml glass bulb (fig. 3), fitted with a Monel valve, is used as a sample holder and absorption vessel. In preparation for analysis the bulb is evacuated to a pressure below 0.01-mm Hg, 1 or 2 ml of mercury are drawn into the bulb, and it is again evacuated and heated to drive off residual gases and water vapor. It is then filled to 1-atm pressure with the gas to be analyzed. Because of surface film formation, no appreciable absorption of fluorine by mercury occurs so that the bulb is readily filled. With the valve closed and continuous violent shaking to break the surface film absorption is complete in 10 to 15 min. with evolution of considerable heat. The end of the reaction is identified by the cooling of the bulb and by a sudden change in appearance of the fluorine film on the mercury from a yellowish or slightly iridescent texture to a dull gray. The residual pressure is measured by an oil manometer as shown in figure 4. The reading of the manometer is corrected for the increase in volume of the gas when admitted to the evacuated manometer arm. By this procedure determination of total impurities up to several percent is possible, though its accuracy is probably limited by the adsorption of some gas in the surface film of mercuric fluoride, and perhaps by the reaction of oxygen difluoride and nitrogen trifluoride under the conditions of the experiment. The reproducibility in a short series of measurements of impurities at the 3-percent level is approximately ±0.05 percent.

When analyzed by this method commercia fluorine shows total impurities of 1 to 3 percent Analysis of the residual gas by mass spectrometer shows it to consist principally of tetrafluoromethane, oxygen, and nitrogen. Hydrogen fluoride is presumed to be present in the fluorine as received, but passage of the fluorine through a sodium fluoride trap is routinely practiced before use, so no analysis was made for this substance. If the sodium fluoride trap is immersed in liquid oxygen, the tetrafluoromethane is removed, nitrogen and oxygen are reduced in amount, and their relative proportions change.

### 2.4. Weighing

Weighing of several types of rather bulky containers is necessary during the experiments. A container of nearly equal volume and weight is used as a tare in order to reduce air buoyancy corrections. Sample bulbs for non condensable gases are spun aluminum or stamped Monel spheres of 0.015-in. to 0.020-in. wall thickness and 3-in. diam, fitted with a 3-in. long tubular neck of the same material as the bulb to permit possible refrigeration, and closed with a small brass valve (see fig. 3). These spheres under a gage pressure of 140 to 150 psi contain up to 0.1 mole of usable gas and weigh about 70 or 140 g depending on the material. Although aluminum bulbs are lighter in weight the difficulty of obtaining aluminum welds free from leaks makes Monel more desirable. Monel also is preferable because of its greater strength. In the aluminum bulbs the joint from aluminum to the brass valve was made successfully by first coating each metal with its appropriate solder, and then joining and finishing with a fillet of aluminum solder.

Condensable gases such as ammonia are retained as liquids in cylinders of heavier wall made of stainless steel and fitted with a similar valve of stainless steel (see fig. 3).

The weighing tube for hydrogen fluoride is a U-tube of Monel and copper, closed at the ends with bolted Monel flanges sealed with Teflon gaskets. The ends are fitted with Monel valves. The U-tube is filled with helium at 1-atm pressure before weighing each time.

### 2.5. Conduct of an Experiment

An experiment is initiated in a wav customary in combustion calorimetry with the calorimeter temperature preset at about 3 deg below the jacket temperature. The observations of temperature of the calorimeter at given times are divided into three periods: an initial period in which changes in temperature are due entirely to thermal leakage and heat of stirring, a middle period in which the principal part of the temperature rise results from the beat produced by the combustion reaction, and a final period in which the temperature change is again due entirely to thermal leakage and heat of stirring. The lengths of the initial and final periods are at least 10 min each, and during these periods all gases but the fuel are flowing in the system. The middle period is started by introducing fuel into the burner. Fuels containing hydrogen generally ignite spontaneously, and the rate of temperature rise due to the reaction can be made nearly constant by keeping the fuel flow rate constant. To conclude combustion the flow of fuel is shut off and replaced by helium flow to purge the line between the fuel sample weighing bulb and burner and thereby cause the combustion of all fuel leaving this bulb. After combustion ceases the rate of temperature rise drops rapidly. The end of the middle period and beginning of the final period take place when the rate of temperature rise diminishes to a constant value.

Time-temperature readings are made at intervals of 1 min during the initial and final periods and at intervals of 0.1° C during the middle period. The time-temperature data are used to correct the observed temperature rise of the calorimeter for heat of stirring and thermal leakage (including heat introduced or removed by the flowing gases) as explained later.

**Figure 5:**
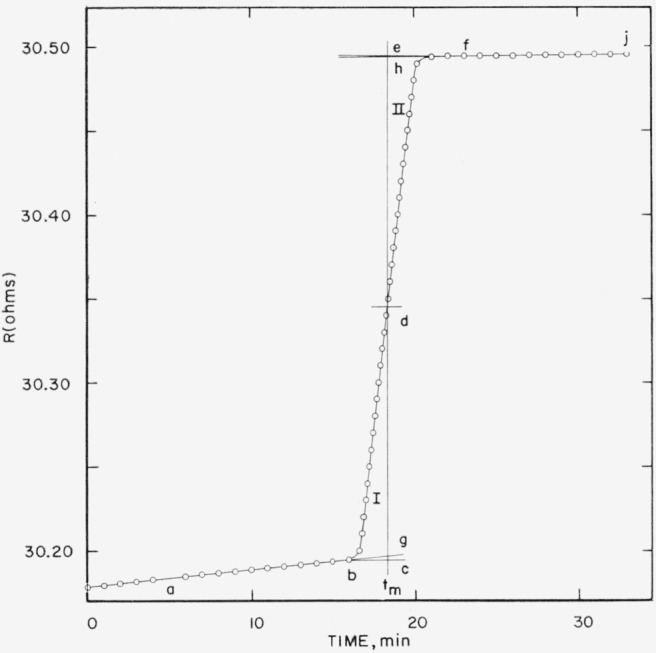
Time-resistance plot for combustion experiment Area *bcd*, (I) equals area *def* (II). Corrected resistance rise is *R_h_−R_g_*.

After the final drift period, fluorine is purged from, the system by helium, leaving the hydrogen fluoride absorption tube filled with helium for weighing. Figure 5, a characteristic time-resistance plot for a fluorine combustion experiment, shows that no obvious anomalous effects occur to disturb the reliability of the measurement of temperature rise.

For work of the accuracy of these experiments the corrected temperature rise is determined by graphically finding a time *t_m_* which causes areas *bcd* and *def* to be equal. The difference between the extrapolated initial and final drift period resistances at *t_m_* is the desired corrected temperature rise [7]. For more accurate work a numerical integration procedure can be used for determining the thermal leakage and the corrected temperature rise [5].

Fuel sample bulb and sodium fluoride trap are weighed before and after the experiment. At some time during the actual combustion, the glass sample bulb is filled with a sample of gaseous products for later analysis if desired. After collection of such a sample, the bulb is first shaken to promote the absorption of fluorine by the mercury. The residual gas is then analyzed by a mass spectrometer.

## 3. Calibrations, Corrections, and Measurements

### 3.1. Calibration

The calorimeter may be calibrated electrically or by means of a chemical reaction of known heat, depending upon the immediate circumstances. The former method is more precise and was used in a study of the methane-fluorine combustion [6]. Satisfactory calibration can be obtained using a four-terminal constantan heating element sheathed in copper, with its leads in close thermal contact with both calorimeter and jacket and with the potential leads attached to the current leads at points halfway between jacket and the point of emergence of the current leads from the calorimeter water. The method of making electrical calibrations has been adequately described by Coops, Jessup, and van Nes [12] and by Rossini [5].

As the result of four determinations, an energy equivalent of 14806.7 j/°C was determined with a standard deviation of the mean of 1.4 j/°C (0.009 percent). The calibration results are shown in table 1, in which *I* is the heater current, *E* is the heater voltage, Δ*R_c_* is the corrected resistance thermometer change, Δ*t* the corrected temperature rise, and E_s_ is the energy equivalent of the calorimeter. The calorimeter to which this calibration applies includes the calorimeter can containing a weighed amount of water, lid, electrical heater, stirrer, thermometer, and the burner assembly, with flow lines attached and filled with stationary air. The calibration was assumed to be the same with stationary air and flowing gases in the burner.

On the basis of later discussions of the combustion of methane and of ammonia, a nonelectrical calibration of the calorimeter can be made with somewhat less precision using either of these reactions. An indication of the precision to be achieved is shown by the standard deviation of the mean heat of formation of HF found as the result of four experiments with ammonia shown in column 7 or column 11 of table 5, or by the standard deviation of the mean calorimeter constant Δ*R_c_*/*m* found in a slightly different calorimeter setup as the result of seven combustion reactions with methane shown in table 4. In table 4 Δ*R_c_* is the corrected temperature rise of the calorimeter caused by burning *m*(CH_4_) grams of methane. The standard deviation of the mean Δ*R_c_*/*m* is 0.32 percent. This estimate of the uncertainty in the calorimeter constant includes both variations among experiments and also uncertainties in the amount of reaction for each experiment as calculated on the basis of observed mass of methane passed through the burner and calculated mass of methane determined from the gain in weight of the hydrogen fluoride absorber.

### 3.2. Correction for Non-Ideality of Hydrogen Fluoride

In a combustion experiment the thermal corrections to the observed heat for gas non-ideality are small for fluorine and for helium. For other gases entering the reaction such thermal effects must be examined carefully. In measurements of reactions involving gaseous hydrogen fluoride, large corrections may be necessary to reduce the results of such measurements to the basis of the ideal gas state; i.e., the standard state of unit fugacity for this gas. Unfortunately, the magnitude of these corrections is subject to considerable uncertainty because of discrepancies between the *PVT* data reported for hydrogen fluoride by various observers [13, 14, 15].

The magnitude of the uncertainty in the correction may be seen by comparing values of (H°−H), the enthalpy of the ideal gas less that of the real gas, as calculated from the data of the two most recent and most extensive investigations of the *PVT* relation of hydrogen fluoride in the pressure range of interest; namely, those of Long, Hildebrand, and Morrell [14] and of Strohmeier and Briegleb [15(a)].

**Figure 6:**
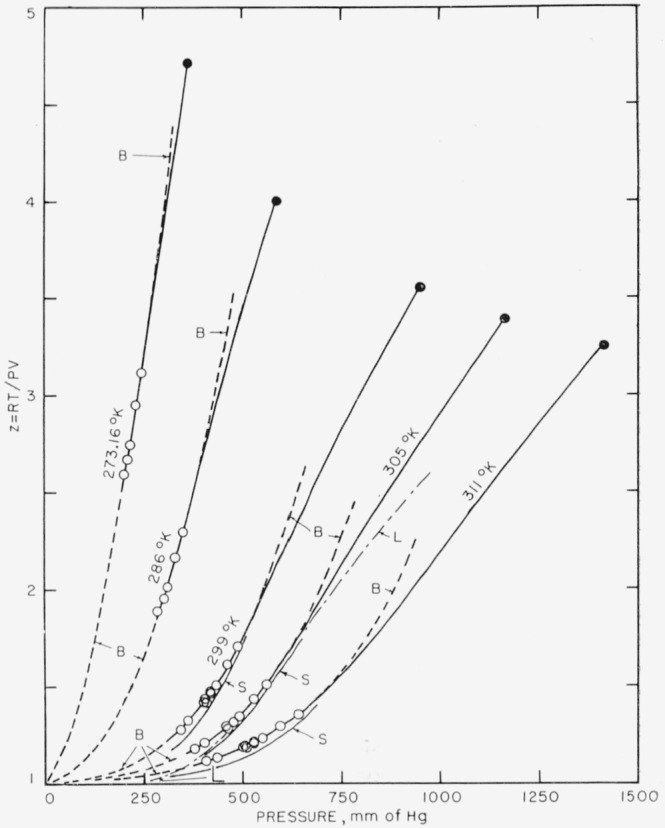
Values of association factor for gaseous HF Open circles, data of reference [14]. Solid circles, data of reference [24]. Solid curves drawn through experimental points. Curves *B* calculated as described in text from data of reference [14]. Curve *L* calculated from data of reference [14], assuming only monomer and hexamer molecules present. Curves *S* represent experimental data of reference [15(a)].

The large deviations of hydrogen fluoride from, ideality have been attributed to the formation of polymers as a result of hydrogen bonding, and two models of the gas have been proposed on this basis. Long, Hildebrand, and Morrell [14] found that their data could be represented fairly well by assuming that the gas is largely a mixture of HF and (HF)_6_, the latter being assumed to be cyclic in structure. The heat of polymerization at a temperature of 305° K and pressure of 0.3 atm, a typical concentration of hydrogen fluoride in the combustion products, as calculated on the basis of this model is entirely negligible in comparison with the measured heat of fluorination of methane, for instance. It will be seen from figure 6, however, that the values of the association factor, *Z=RT/PV*, calculated for this model (curve *L*) fall considerably below the experimental values at low pressures, so that the model does not give a correct representation of the *PVT* data at low pressures. This fact was observed by Long et al. [14], who suggested that under conditions in which the association factor is less than 1.3 lower polymers play an appreciable role.

The other model of hydrogen fluoride, originally proposed by Briegleb [16], assumes that the gas is a mixture of polymers (HF)*_n_* in which *n* assumes an indefinite number of integral values. Evidence favoring this model includes electron diffraction data [17] which indicate the presence of chain polymers, and measurements of the dielectric constant of the gas [18] which show that its dipole moment increases with pressure, contrary to what would be expected if the polymer exists solely in the ring form.

Considering all the data available, it appears probable that both linear and cyclic polymers are present in the gas phase. However, because only the low pressure region is of interest in this work, a model similar to that of Briegleb has been assumed in making calculations of (H°−H). Using this model and the experimental association factors of Long, Hildebrand, and Morrell [14], equilibrium constants for the polymerization reactions

$$2\text{HF}\to {\left(\text{HF}\right)}_{2}$$

and

$${\left(\text{HF}\right)}_{n}+\text{HF}\to {\left(\text{HF}\right)}_{n+1}\;(n=2,3,\ldots )$$

were derived on the assumption that the equilibrium constant *k_n,n_*_+1_ for the second reaction is independent of *n*, and the further assumption that deviations from ideality are due entirely to polymerization. The equilibrium constants obtained from the experimental data are compared in table 2 with values calculated from the following empirical equations

$$-R\;\ln\;k_{1,2}=-\frac{15488.5}{T}+55.2631,$$

$$-R\;\ln\;k_{n,n+1}=-\frac{1329.6}{T-169.92}+9.6588,\;(n>1),$$

where *R* is in cal/mole deg K and pressures are expressed in atm.

The equilibrium constants were used to calculate values of the association factor and the results of these calculations are shown by the curves marked *B* in figure 6. In the pressure ranges covered by the experimental data of Long, Hildebrand, and Morrell these curves are seen to be practically identical with the solid curve drawn through the open circles representing their data and the solid circles representing the data of Jarry and Davis [19] at saturation pressure. Between these two sets of points, however, the curves *B* begin to diverge from the experimental curves. Nevertheless the curves *B* seem to give a satisfactory interpolation between the pressures covered by Long, Hildebrand, and Morrell and zero pressure, where the gas is presumed to be ideal and monomeric. It will be seen that the curve *B* for *T*= 305° K fits the experimental points of Long, Hildebrand, and Morrell at low pressures better than does their curve *L* derived on the basis that only monomer and hexamer are present.

Using the temperature coefficients of the equilibrium constants, the heats of the polymerization reactions were calculated, and these together with the association factors were then used to calculate values of (H°−H) per mole of HF.

The data of Strohmeier and Briegleb [15(a)] have also been used to calculate values of (H°−H), using the equilibrium constants and their temperature coefficients reported by Briegleb and Strohmeier [15(b)] for the reactions

$$n\text{HF}(g)={\left(\text{HF}\right)}_{n}(g)\;(n=2,3,\ldots ).$$

The association factors calculated from these equilibrium constants have been shown by Briegleb and Strohmeier (15(b)] to be in very good agreement (about 0.1 percent) with their experimental values. The curves calculated by Briegleb and Strohmeier to represent association factor versus pressure (experimental points not shown) for the three isotherms covered both by Long, Hildebrand, and Morrell, and by Strohmeier and Briegleb are those marked *S* in figure 6. The two sets of curves differ systematically by considerably more than would be expected from the precision of the two sets of measurements.

**Figure 7:**
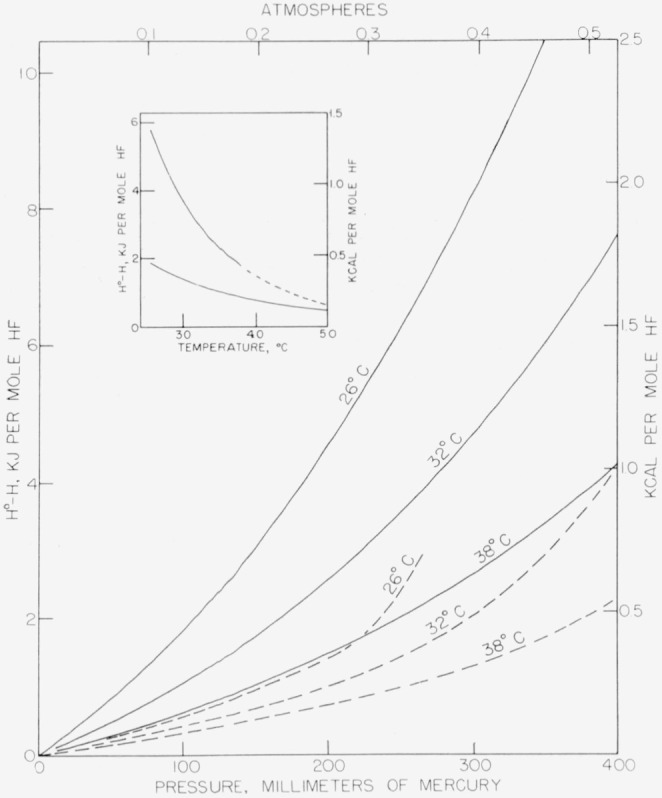
Change in enthalpy with pressure for hydrogen fluoride at 26°, 32°, and 38° C Solid curves calculated as described in text from data of reference [14]. Dashed curves calculated from data of reference [15(a), 15(b)]. Insert: Isobar at 0.3 atm calculated from data of reference [14] (upper curve) and from data of reference [15(a), 15(b)] (lower curve).

Values of (H°−H) calculated from both sets of data are given in table 3 and are shown graphically in figure 7. The values based on the data of Long, Hildebrand, and Morrell at temperatures above 38° C represent extrapolations beyond the range of their experimental data. The values calculated from the data of Long, Hildebrand, and Morrell are higher by a factor of two to three than those calculated from the data of Strohmeier and Briegleb.

As an indication of the possible effect of these discrepancies we may consider the measurements of Jessup, McCoskey, and Nelson [6] who determined the heat of formation of CF_4_ from the measured heat of reaction of methane and fluorine combined with values for the heats of formation of methane and hydrogen fluoride. Under the conditions of their experiment the correction for the gas imperfection of HF calculated from the data of Long, Hildebrand, and Morrell, was 2.1 kj (0.5 kcal) per mole of HF, or 8.4 kj (2.0 kcal) per mole of CF_4_. If the data of Strohmeier and Briegleb had been used, the correction would have been only 3.3 kj (0.8 kcal) per mole of CF_4_, so that the value obtained for the heat of formation of CF_4_ would have been different by 5.1 kj (1.2 kcal) per mole.

In view of the discrepancies between the data reported in reference [14] and [15], further experimental work on the gas imperfections of hydrogen fluoride would be very desirable.^3^ Such work might include further *PVT* measurements, throttling experiments, or calorimetric experiments similar to those reported by Rossini and Frandsen [20].

Until the *PVT* behavior is known with greater certainty in the low pressure region at temperatures near 25° C, the problem of corrections to calorimetric data can be avoided by making measurements at higher temperatures. The work of Wartenberg and Fitzner [1] at 100° C, for example, does not suffer from any such uncertainty because the enthalpy corrections are negligible for hydrogen fluoride at that temperature. It will also be seen by reference to the 0.3-atm isobar in figure 7 (insert) that in the neighborhood at 50° C the differences between the experimental data become negligible in this isobar which is typical. In some experiments at NBS measurements have been made at 50° C taking advantage of this fact.

### 3.3. Measurement of Amount of Reaction

The amount of fuel entering the calorimeter is readily determined by weighing the container before and after the experiment. The early measurements in the fluorine flame calorimeter suffered from lack of confirmation that the entering fuel was completely burned. The vapor pressure of HF over the compound NaF·HF is very small at room temperature (about 0.01-mm Hg [21]). The use of an absorbing tube to collect for weighing the hydrogen fluoride resulting from the combustion of hydrogenous fuels is thus an obvious technique for verifying the completeness of reaction, and has been used throughout these experiments. This absorbent has also been used by Wartenberg and Schutza [2], though not in the same manner as here. They may not have encountered the same difficulties because of the use of hydrogen as the gas in excess rather than fluorine. In none of the early experiments in this laboratory, either in the combustion of methane or of ammonia, was consistent correlation found between weight of fuel and weight of hydrogen fluoride formed. The most general behavior was for the weight of hydrogen fluoride to be rather erratically too high.

The excess weight gain was traced (a) to impurities (HF and perhaps some CF_4_) in the fluorine supply, and (b) to additional impurities (presumably HF) acquired by the fluorine first passing through the combustion chamber. Impurities in the fluorine supply may be removed by allowing the fluorine to pass through a sodium fluoride trap immersed in a dry-ice-alcohol bath just before entering the calorimeter. Collection and weighing of the added impurities acquired by the fluoride in its passage through the combustion chamber may be avoided by diverting the fluorine through a valve bypassing the hydrogen fluoride weighing tube until time for introduction of the fuel. Evidence for the nature of the impurities to be avoided in this way is as follows. After a combustion reaction, the inner surface of the copper combustion chamber has a brick-red color on first opening for inspection. On standing, the red color changes to white, a behavior which is attributed to the hydrolysis of red cuprous fluoride by moisture in the air. In preparing for the fore-period drift measurements, the first introduction of fluorine always causes a sharp increase in the drift rate, which disappears after a few minutes leaving the drift rate approximately the same as in the absence of fluorine. By blank experiments in which the calorimeter was not opened to the air it was found that increases in the weight of the hydrogen fluoride weighing tube cease a few minutes after the first introduction of fluorine. The gain in weight could thus be attributed to gases formed by interaction of fluorine with substances on the surfaces of the combustion chamber.

Table 4a shows a typical series of weighings in calorimetric experiments with methane, using experiments in which no carbon formation was observed, and in which all of the gases used in the experiment passed through the hydrogen fluoride weighing tube. In this table *m*(CH_4_) is the weighed mass of methane passed through the burner, and *m*′(CH_4_) is the mass of methane calculated on the basis of the weight of hydrogen fluoride collected. The mean difference is 2.1 percent. In the first three columns of table 4b are shown data for a series of experiments in which gases were collected in the hydrogen fluoride weighing tube only after the initial drift period was nearly complete. In these experiments the mean difference between *m*(CH_4_) and *m*′(CH_4_) without regard for sign is 0.13 percent, a significant improvement over the preceding series of experiments.

By these experiments it has thus been shown possible to confirm the amount of reaction of a hydrogenous fuel with reasonable accuracy by relatively simple precautions in the conduct of an experiment. Some uncertainty remains because of the present inability to collect other product gases for weighing or analysis, and a sampling technique must be resorted to for the indication of side reactions. In the specific instances of the combustion of methane and ammonia the reactions have been found to be simple and not accompanied by measurable amounts of side reactions.

## 4. Heat of Reaction of Ammonia With Fluorine

The combustion of ammonia in fluorine was found to proceed without the formation of any fluorides of nitrogen when carried out in the burners described, contrary to the experience of Ruff and Hanke [22]. Absence of compounds of nitrogen and fluorine in the product gases was determined by mass spectrometer. The only reaction observed was that shown by eq (1)

$${\text{NH}}_{3}+\frac{3}{2}F_{2}\to 3\text{HF}+\frac{1}{2}N_{2}$$ (1)

The results of four combustion reactions carried out between 30 and 32° C are shown in table 5. The amount of reaction was based on the weight of ammonia burned (col. (1)) because the collection of HF was not under control in these experiments. The observed heat of reaction is shown in column (2); the partial pressure of hydrogen fluoride in the effluent gases, determined by the proportions of materials introduced is shown in column (3). Column (4) shows the correction for non-ideality of hydrogen fluoride calculated on the basis of the data of Long et al. by application of which are calculated the standard heat of reaction, column (5), and the standard heat of formation of hydrogen fluoride, column (6). Column (8) shows the correction for non-ideality of hydrogen fluoride calculated on the basis of the data of Strohmeier and Briegleb, by application of which are calculated the standard heat of reaction shown in column (9) and the standard heat of formation of hydrogen fluoride, column (10). Columns (7) and (11) give the deviation from the mean, *δ*, of the individual results in columns (6) and (10), respectively. In the above calculations a correction of −0.13 kj/mole is made for the difference $\Delta H_{25}^{{}^{\circ}}-\Delta H_{32}^{{}^{\circ}}$. The heat of formation of ammonia is well established and was taken as −46.19 kj/mole= −11.04 kcal/mole [23]. The mean heat of formation of HF in column (6) is −268.7±0.5 kj/mole= −64.22±0.13 kcal/mole, in which the uncertainty given is the standard deviation of the mean. This may be compared with −64.2 kcal/mole found in reference [23], which is an average based on values of −63.8 kcal/mole calculated by Ruff and Menzel [3] from the experimental work of Wartenberg and Fitzner [1], −64.2 kcal/mole calculated by Ruff and Menzel from the experimental work of Ruff and Laass [24], and −64.45±0.1 kcal/mole reported by Wartenberg and Schutza [2].

However, the average value of column (10) is −270.4±0.3 kj/mole= −64.63±0.07 kcal/mole. The scatter is less in this calculation than in the preceeding one, but it is obvious that the principal source of uncertainty is in the amount of correction to be applied for non-ideality of hydrogen fluoride. The mean of all the calculated values gives $\Delta H{{}^{\circ}}_{f_{25}}(\text{HF})=-269.6\pm 1.1\;\text{kj}/\text{mole}=-64.4\pm 0.25\;\text{kcal}/\text{mole}$, as the best value which can be derived from the data presented here.

It might be noted that several values [6, 25, 26] for the heat of formation of carbon tetrafluoride are based in part on the value −64.2 kcal/mole for the heat of formation of HF. A more negative value for the heat of formation of hydrogen fluoride would tend to bring closer together the values for heat of formation found on the basis of oxygen bomb calorimetry [25, 26] on the one hand and by fluorine flame calorimetry [6] on the other hand.

## Figures and Tables

**Table 1:** Electrical calibration of the calorimeter

Run	Time	*I*	*E*	*Q*	Δ*R_e_*	Δ*t*	E*_g_*
							
	*sec*	*amp*	*v*	*j*	*Ohm*	*deg C*	*j/deg C*
1	660.00	2.63371	25.6401	44568.9	0.303295	3.00924	14810.7
2	660.00	2.63181	25.5966	44461.2	.302289	3.00279	14806.6
3	660.00	2.65606	25.8362	45290.9	.308391	3.05883	14806.5
4	660.00	2.65022	25.7808	45094.4	.306762	3.04628	14803.1

Mean	14806.7±1.4 (0.009%)						

**Table 2:** Observed and calculated values of equilibrium constants derived from data of reference [14]

Temperature	*k*_12_		*k_n,n_*_+1_	

*°K*	*obs.*	*calc.*	*obs.*	*calc.*
311	0.0764	0.0641	0.862	0.889
305	.1050	.1049	1.100	1.097
299	.1748	.1751	1.375	1.381
286	.5730	.5927	2.477	2.469
273.15	2.0620	2.0619	5.050	5.056

**Table 3:** Enthalpy differences (H°−H) for hydrogen fluoride gas

	H°−H kj/mole				H°−H kcal/mole			
	a. Calculated to fit the data of Long, Hidebrand, and Morrell							

*T* deg C	26	32	38	50	26	32	38	50
*P* atm								

0.1	1.32	0.77	0.46	0.18	0.316	0.185	0.110	0.043
.2	3.10	1.78	1.05	.38	.742	.426	.250	.091
.3	5.49	3.10	1.77	.63	1.312	.740	.424	.150
.4	8.43	4.80	2.71	.91	2.015	1.146	.647	.218
.5	11.89	6.95	3.89	1.26	2.842	1.660	.930	.300

	b. Calculated to fit the data of Strohmeier and Briegleb									

*T* deg C	26	32	38	44	56	26	32	38	44	56
*P* atm										

0.1	0.41	0.32	0.25	0.19	0.12	0.098	0.076	0.059	0.046	0.028
.2	.97	.70	.53	.40	.24	.232	.167	.126	.096	.058
.3	1.81	1.23	.86	.63	.37	.432	.293	.205	.150	.088
.4	………	2.11	1.31	.90	.50	………	.502	.314	.216	.120
.5	………	………	2.02	1.25	.66	………	………	.483	.299	.158

**Table 4:** Consistency of weighings and reproducibility of heat measurement in the combustion of methane by fluorine

a. All gases pass through hydrogen fluoride trap			
*m*(CH_4_)	*m*′(CH_4_)	*m*(CH_4_)*−m*′(CH_4_)	Difference

*g*	*g*	*g*	%
0.4139	0.4215	−0.0076	−1.8
.4020	.4075	−.0055	−1.4
.4020	.4173	−.0153	−3.8
.4074	.4129	−.0055	−1.3

b. Fore-period gases do not pass through hydrogen fluoride trap						
*m*(CH_4_)	*m*′(CH_4_)	Difference	Δ*R_c_*	$\frac{\Delta R_{c}}{m({\text{CH}}_{4})}$	$\frac{\Delta R_{c}}{m^{\prime }({\text{CH}}_{4})}$	*t*

*g*	*g*	%	*Ohm*	*Ohm/g*	*Ohm/g*	° *C*
0.3923	0.3925	−0.05	0.31090	0.7925	0.7921	49.75
.3940	.3938	+.05	.31362	.7960	.7964	49.77
.4053	.4068	−.37	.31964	.7887	.7857	49.72
.3987	.3986	+.02	.31558	.7915	.7917	49.71
.4031	.4036	−.12	.32130	.7971	.7961	49.72
.4138	.4131	+.17	.32851	.7939	.7952	49.69
.4335	.4341	−.15	.33745	.7784	.7773	49.86

Mean		±0.13	………	0.7912	0.7906	………

Mean: Δ*R_c_/m*, Δ*R*_c_/*m*′ =0.7909±0.0025

**Table 5:** Combustion of ammonia in fluorine

(1) *m*(NH_3_)	(2) ΔH_32_ obs	(3) *p*(HF)	(4) *q*(HF)(1)	(5) ΔH°_25_(1)	(6) $\Delta H{{}^{\circ}}_{f_{25}}(\text{HF})$(1)	(7) *δ*(1)	(8) *q*(HF)(2)	(9) ΔH°_25_(2)	(10) $\Delta H{{}^{\circ}}_{f_{25}}(\text{HF})$ (2)	(11) *δ*(2)

*g*	*kj/mole* NH_3_	*atm*	*kj/mole* NH_3_	*kj/mole*	*kj/mole*	*kj/mole*	*kj/mole* NH_3_	*kj/mole*	*kj/mole*	*kj/mole*
0.933	−765.7	0.11	2.4	−763.4	−269.9	+1.2	1.0	−764.8	−270.3	−0.1
.8273	−767.6	.31	11.7	−756.0	−267.4	−1.3	4.3	−763.4	−269.9	−.5
.9232	−772.4	.35	14.2	−758.3	−268.2	−.5	5.2	−767.3	−271.2	+.8
.5430	−766.3	.18	5.1	−761.3	−269.2	+.5	2.0	−764.4	−270.2	−.2

Mean (kj/mole)				−759.7	−268.7	±0.55^a^	………	−765.0	−270.4	±0.28^a^
Mean (kcal/mole)				−181.6	−64.22	±0.13^a^	………	−182.8	−64.63	±0.07^a^

a Standard deviation of the mean.

## Footnotes

2 Figures in brackets indicate the literature references at the end of this paper.

3 At least two investigations of the PVT relation of HF are known to be in progress, but cover a pressure range much higher than that of interest in most calorimetric measurements.
